# Outcomes following the operative treatment of intra-articular fracture combined with medial patellofemoral ligament reconstruction after patellar dislocation

**DOI:** 10.1186/s43019-022-00150-6

**Published:** 2022-04-13

**Authors:** Jussi P. Repo, Mikko M. Uimonen, Mika T. Nevalainen, Heikki Nurmi, Ville T. Ponkilainen, Antti Tuominen, Juha Paloneva

**Affiliations:** 1 Department of Orthopaedics and Traumatology, Unit of Musculoskeletal Surgery, Tampere University Hospital, Tampere, Finland; 2 Department of Surgery, Central Finland Hospital, Jyväskylä, Finland; 3 Oulu University Hospital & University of Oulu, Oulu, Finland; 4 University of Eastern Finland, Kuopio, Finland

**Keywords:** Patella, Dislocation, MPFL, Reconstruction, Fracture, Knee

## Abstract

**Purpose:**

We examine the outcomes following operative treatment of intra-articular fracture combined with medial patellofemoral ligament (MPFL) reconstruction after patella dislocation.

**Methods:**

Patients were retrospectively identified from medical records using diagnostic and surgical procedure codes. Radiological anatomical parameters and bony abnormalities of injured knees were assessed from magnetic resonance images (MRI). Inclusion criteria were traumatic patellar dislocation with chondral or osteochondral fracture and MPFL rupture, operative treatment of a chondral or osteochondral fracture combined with MPFL reconstruction, and minimum follow-up of 2 years. Outcomes were measured using the Kujala score, Tegner activity scale, and the Knee injury and Osteoarthritis Outcome Score Quality-of-Life subscale (KOOS-QLS).

**Results:**

During 2012 and 2015, 322 patients were treated because of patellar dislocation. Thirty-three patients had chondral or osteochondral fracture. Eleven patients (five males and six females) with a mean [standard deviation (SD)] age of 17.0 (6.5) years at the time of surgery met the inclusion criteria and were included. Five of the 11 patients had a subchondral and six an osteochondral fracture. Eight patients had a fracture in the patella and three in the femur. All patients had bony abnormalities in the knee. Nine out of 11 patients scored over 90/100 points on the Kujala scale and had good results on the Tegner scale [before surgery 5.0 (2.7) points versus after surgery 5.3 (1.6) points] and the KOOS-QLS [4.1 (4.2) points] outcome measures.

**Conclusion:**

The removal or fixation of the fracture fragment combined with MPFL reconstruction is a feasible option in the treatment of symptomatic osteochondral or subchondral fragment in traumatic patellar dislocation. The short-term outcomes are encouraging.

*Level of evidence:* Level IV, retrospective case series.

## Introduction

Incidence of patellar dislocation surgery is approximately 9 per 100,000 person-years [1]. The first primary traumatic patellar dislocation is usually treated nonoperatively [2], although there are exceptions in cases of large or symptomatic intra-articular fractures [3]. Osteochondral fractures (OCFs) are located in the patella (63%), the lateral femoral condyle (34%), or both (3%) [4]. OCFs larger than 1 cm^2^ have been reported to occur in 14% of traumatic patellar dislocations in children [5]. Furthermore, it has been indicated that cartilage damage of more than 1 cm^2^ can increase degeneration of the cartilage [6]. In addition, patients with or without minor bony malformations related to patellar instability may be at increased risk for OCF after patellar dislocation [7]. The published literature on the clinical outcomes of the treatment of patellar instability with chondral of osteochondral fractures is scarce. Currently, subchondral laminar or osteochondral intra-articular fractures can be treated by either removal or fixation of the fracture fragment [5, 6]. The techniques of microfracture, subchondral drilling, and periosteal or abrasion chondroplasty have also been proposed [8]. Furthermore, medial patellofemoral ligament (MPFL) reconstruction can be combined with the treatment of intra-articular fractures [5].

To the the best of the authors’ knowledge, there is little evidence of the impact of the combined treatment of intra-articular fractures and MPFL injury on outcomes. Therefore, this study retrospectively investigated the outcomes of the treatment of patellar instability with fixation or removal of symptomatic chondral or osteochondral fracture with fracture fixation and MPFL reconstruction after first or recurrent patellar dislocation. The recurrence of patellar dislocation after this combined surgical treatment was also investigated. It was hypothesized that complications would be rare, there would be no recurrent dislocation after surgery, and postoperative patient-reported outcomes would be good.

## Materials and methods

The institutional review board (IRB) of Central Finland Healthcare District (CFHD) approved the study protocol. The study was register-based, which does not need ethical approval according to Finnish law (Medical Research Act, 488/1999)” (https://www.finlex.fi/en/). CFHD granted permission for the study, which was performed at Central Finland Hospital (CFH), Jyväskylä, Finland. CFH is a public hospital providing traumatological treatment to a catchment population of 252,000, or approximately 5% of the population of Finland. The electronic hospital discharge register was searched for the years 2012–2015, using ICD-10 codes S83.0 (Patellar dislocation) and M22.0 (recurrent patellar dislocation), and the Nordic Medico-Statistical Committee (NOMESCO, Finnish version) classification of surgical procedure codes NGF20, NGF30, and NGF35 (NGF20, operation for osteochondritis of knee, open; NGF30, fixation of loose body of joint of knee; NGF35, plastic operation of joint cartilage of knee). Patient records were retrospectively retrieved from an electronic patient record system (Effica, Tieto Corporation, Helsinki, Finland). Indication for MPFL reconstruction was (1) normal J-sign and recurrent patellar dislocation, or (2) OCF when MPFL reconstruction was always performed together with the surgical treatment of OCF. If the J-sign was positive, bony procedures such as distalization of the tibial tubercle were added to the MPFL reconstruction.

**Fig. 1:**
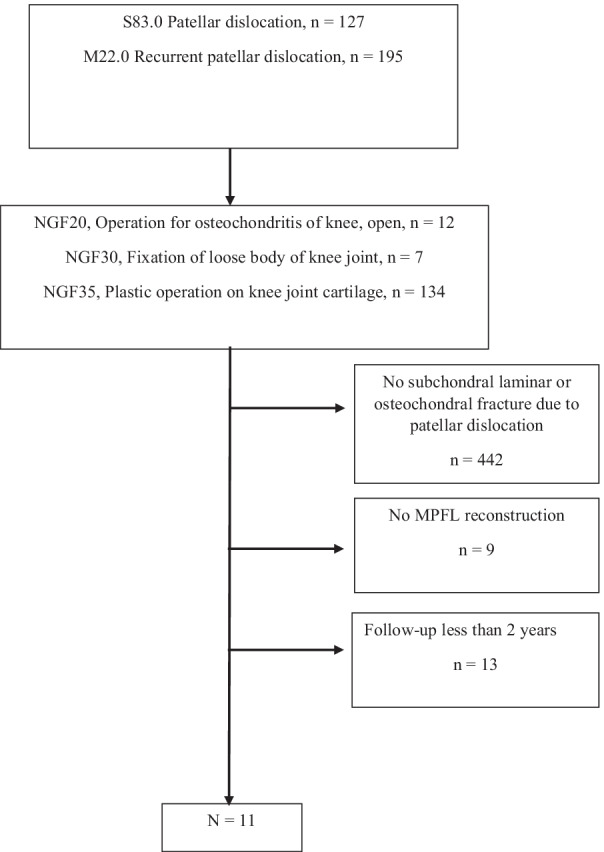
Flow chart of patient selection

Inclusion criteria for the study were traumatic patellar dislocation, symptomatic chondral or osteochondral fracture and MPFL rupture, operative treatment of chondral or osteochondral fracture combined with MPFL reconstruction, and completion of the follow-up outcome measures at least 2 years after surgery. A flow chart of patient selection is shown in Fig. 1. The Strengthening the Reporting of Observational Studies in Epidemiology (STROBE) guidelines were adhered to in the reporting of the results of this study.

### Operative treatment

Fixation of osteochondral or subchondral laminar fractures was performed with biodegradable pins (SmartPin, Bionx Implants Ltd, Tampere, Finland). The fragment was fixed if the size of the defect was larger than 1 cm^2^ in size and located on the weight-bearing surface of the knee or facet of the patella. If the fragment was fragmented from the non-weight-bearing surface of the knee, it was removed through arthroscopy or with open surgery. If periosteal arthroplasty was performed, periosteum from the tibia was used. In addition, microfractures were performed in some patients. The MPFL was reconstructed using adductor magnus, gracilis, or semitendinosus tendon grafts. The graft ends were attached with 2–0 Vicryl sutures (Ethicon Inc., Somerville, New Jersey, Ohio, USA) by supporting the distal part of the graft and tapering the ends. Two tunnels were drilled obliquely through the patella from the medial facet to the anterior side of the medial patella. The graft was then threaded through the tunnels, forming a loop as the graft passed through one tunnel and emerged from the other. In the medial epicondyle, a direct socket was drilled into the condyle, keeping to the optimal isometric point with the help of C-arm radiography. Flexion and extension movements were also performed to determine the isometric point of the patella. When required, the socket was placed under the growth plate, thereby keeping the growth plate intact and supporting immature bone growth. The graft was attached to the socket with a biodegradable headless Milagro interference screw (Milagro Advance, DePuy Synthes, Raynham, Massachusetts, USA).

### Postoperative treatment protocol

Full weight-bearing on a straight leg and free range of motion were allowed immediately after the operation. Stair-walking was to be avoided for 6 weeks. Riding a bicycle and minor squat exercises began at 6 weeks. Closed kinetic chain exercises (hack squat and leg press) with progressively increasing force were recommended from 6 weeks to 2 months. Squat exercises with light additional weights and jogging were recommended after 3 months. Unlimited exercise and movements were allowed from 4 to 6 months postoperatively.

### Radiological measures

Radiological parameters of the knee joint were assessed using preoperative native X-ray images of the knee in 30° flexion and MRI (1.5 T) of the knee joint in full extension. The location and size of the subchondral laminar or osteochondral fracture and the presence and location of MPFL injury were examined using MRI. The three-dimensional diameters (height, width, and depth) of the loose fragment were measured. The size of the articular surface area of the fragment was estimated by multiplying the longest dimension by the second longest. Trochlear dysplasia was assessed according to the Dejour dysplasia classification using MRI [9, 10].

The Insall–Salvati index (ISI), Caton–Deschamps index (CDI), and patellotrochlear index (PTI) were measured from X-ray images to assess the vertical location of the patella in relation to the trochlea [11–13]. The ISI is calculated by dividing the length of the patellar tendon by the height of the patella [11]. The CDI is calculated by dividing the distance between the lowest patellar edge and the highest tibial edge by the length of the patellar articular surface [12]. Patellar height was defined as pathological, i.e., as indicating patella alta [11, 12, 14], when the ISI value was over 1.2 and the CDI value over 1.3. The patellotrochlear index (PTI) is an alternative tool for evaluating patella height [15]. The PTI is calculated by dividing the distance between the highest and the lowest points of the trochlear cartilage surface (*A*) by the distance between the highest and the lowest points of the patellar cartilage surface (*B*) [15]. The result is multiplied by 100% (*A*/*B* × 100% = PTI). A normal value of the PTI is over 50%, a ratio under 15% indicates patella alta, and values between these represent a gray area [20, 22].

To assess lateralization of the patella, tibial–trochlear groove (TT-TG) distance, and tibial tubercle–posterior cruciate ligament (TT-PCL) [11] distance were measured using MRI. Normal values for TT-TG in a pediatric population with a normal patella have been proposed to range between 8.9 mm and 11.1 mm [16], and values over 12 mm are considered to be pathological [17]. For TT-PCL, normal values are considered to be less than 16.6 mm and values over 20 mm pathological [18, 19].

The current situation of the knee at follow-up was assessed using the Kujala score, the Tegner Activity Scale, and the Health-Related Quality of Life section of the Knee injury and Osteoarthritis Outcome Score (KOOS) [20–22]. Recurrent patellar dislocations and reoperations were examined until the last follow-up date. The results are presented as counts or measured values or as means together with standard deviation (SD).

## Results

In total, 322 patients with patellar dislocation between 2012 and 2015 were identified using the diagnostic codes for primary or recurrent patellar dislocation and procedure codes of open debridement, removal of loose fragments of joint cartilage of knee, fixation of loose body of joint cartilage of knee, and chondroplasty of joint cartilage of knee. Thirty-three patients had a subchondral laminar or osteochondral fracture due to patellar dislocation. Eleven patients with a mean (SD) age of 17.0 (6.5), five males and six females, met the inclusion criteria (Fig. 1). The patients’ demographic and clinical details are presented in Tables 1 and 2. Mean follow-up was 4.7 (1.3) years.

**Table 1:** Participants’ demographic and clinical details

	*N* = 11
Age, years, mean (SD)	17 (6.5)
Male, *n*	5
Time between patellar dislocation and operation, days (SD)	22.4 (25.6)
Management of loose fragments, *n*	
Fixation with pins	7
Removal	4
Microfracture to enhance bone regeneration, *n*	2
Periosteal arthroplasty	1
MPFL reconstruction graft, *n*	
Gracilis	7
Adductor magnus	3
Semitendinosus	1

**Table 2:** Sociodemographic details, type of dislocation (first/recurrent), and results of anatomical measurements from radiological images

Patient number	Age at surgery (years)	Gender	First/recurrent patellar dislocation #	ISI	CDI	PTI (%)	Dejour classification	TT-TG (mm)	TT-PCL (mm)	Fracture type	Fracture location	Fracture width (mm)	Fracture height (mm)	Fracture depth (mm)
1	15.4	M	2	1.39	1.48	34	B	7.5	22.1	Subchondral	Femur	24.9	16.9	8.1
2	16.5	M	1	1.09	1.34	77	B	16.4	20.4	OCF	Patella	10.4	6.8	6.1
3	27.3	F	1	1.21	1.41	57	A	6.3	15.2	Subchondral	Patella	18.6	25.4	6.3
4	13.0	M	1	0.96	1.25	71	B	17.1	23.7	OCF	Patella	13.3	9.5	3.7
5	20,4	F	1	1.30	1.79	39	B	15.4	21.6	Subchondral	Patella	11.7	15.6	4.5
6	14.3	M	2	1.21	1.87	57	B	22.1	22.6	OCF	Femur	10.8	19.6	3.6
7	16,6	F	1	1.00	1.17	62	B	17.1	25.3	Subchondral	Patella	23.3	22.3	3.9
8	21.9	F	1	1.13	1.24	37	A	13.6	24.8	OCF	Femur	22.6	16.9	3.5
9	15.7	F	2	1.25	1.34	27	B	19.1	20.4	Subchondral	Patella	20.4	12.6	10.9
10	11.6	F	1	1.51	1.56	72	A	12.4	20.6	OCF	Patella	13.3	20.4	6.9
11	15.1	M	1	1.32	1.45	78	A	10.2	21.6	OCF	Patella	6.9	12.5	5.0

*ISI* Insall–Salvati index, *CDI* Caton–Deschamps index, *OCF* osteochondral fracture, *PTI* patellotrochlear index, *TT-TG* tibial tubercle–trochlear groove distance, *TT-PCL* tibial tubercle–posterior cruciate ligament distance, *M* male, *F* female; # 1 = first patellar dislocation, 2 = recurrent patellar dislocation

**Fig. 2:**
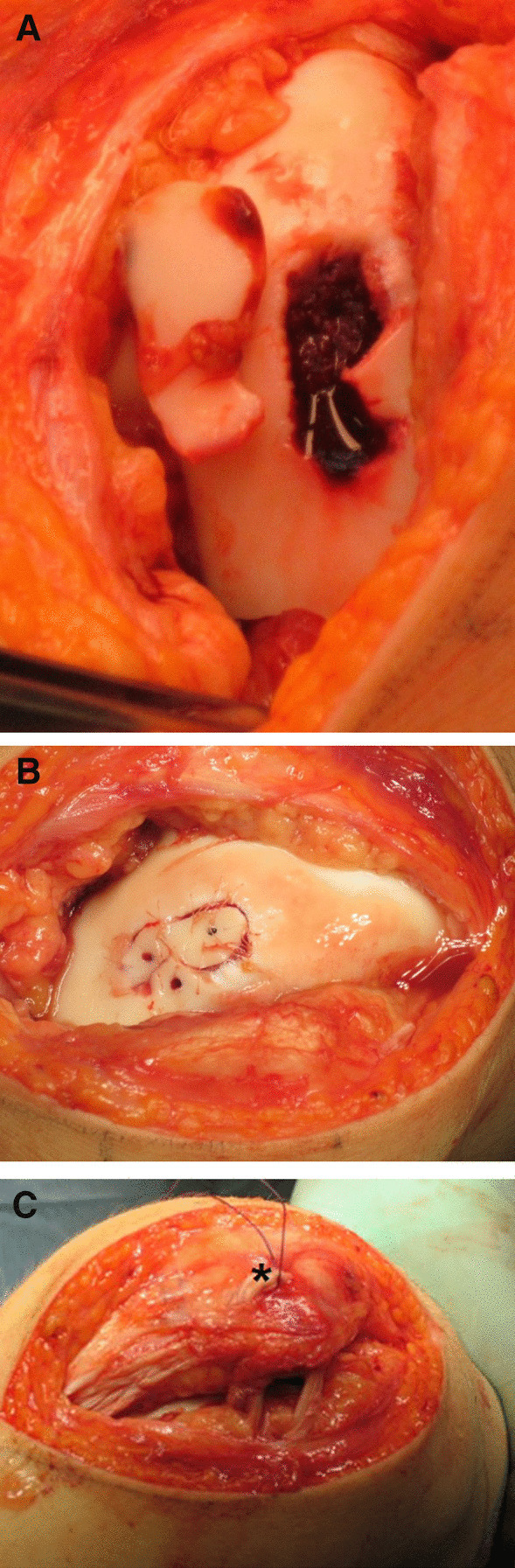
**A** An intraoperative photograph of a large osteochondral fracture sustained by a 14-year-old boy. **B** A photograph showing osteochondral fracture fixation using rods and resorbable sutures. **C** MPFL reconstruction was performed using the adductor magnus tenodesis technique (*). The abductor magnus runs just under the periosteum on the medial side of the patella. It has been attached to the soft tissue with a suture

A subchondral laminar fracture was found in five patients and an osteochondral fracture in six patients (Fig. 2A–C). Eight patients had intra-articular fragments detached from the patella, and three patients had intra-articular fragments detached from the lateral condyle of the femur. The mean (SD) width, height, and depth of the fragments were 13.1 (5.1), 18.9 (5.2), and 5.9 (2.7) mm, respectively. Mean fragment surface area was 2.7 (1.6) cm^2^.

**Fig. 3:**
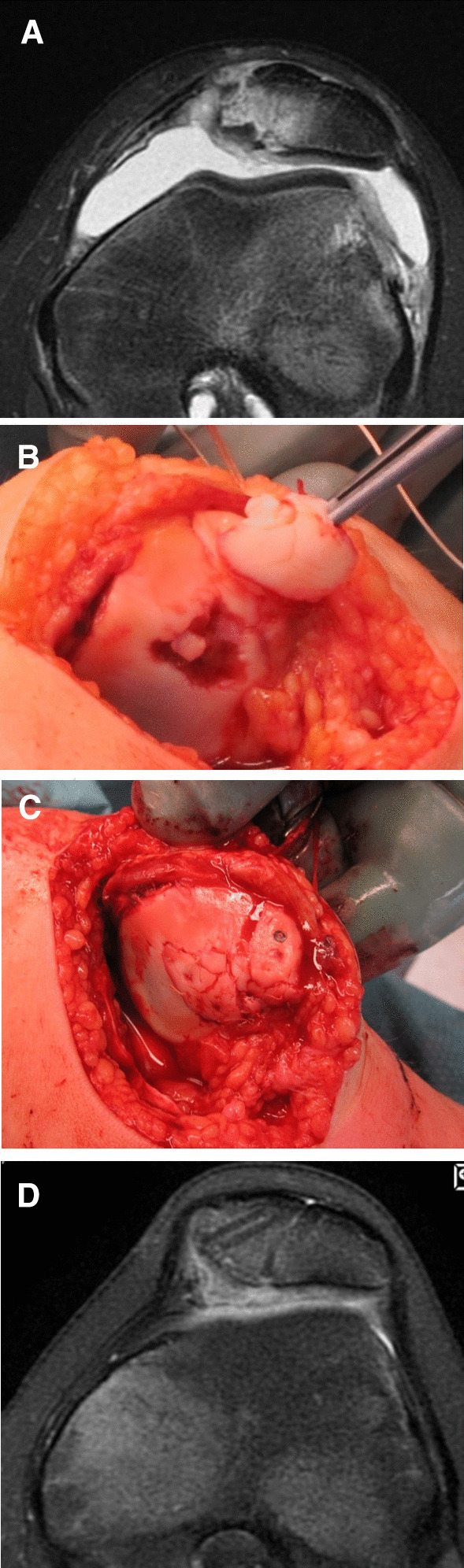
**A** Preoperative MRI of an osteochondral fracture on the medial facet of the patella in a 12-year-old girl who had sustained a patellar dislocation. **B** An intraoperative photograph of two large osteochondral fractures in the same patient. **C** Fixation of osteochondral fractures using rods and bioabsorbable sutures. An MPFL reconstruction was subsequently performed. **D.** Transverse MRI at 1 year follow-up of the same patient as in Fig. 3A–C aged 13 years showing the attached osteochondral fragments in place and the two tunnels where the ligament graft had been located. She had a mild J-sign, but a negative apprehension sign. There was no swelling of the knee, but some crepitus in forced extension. The knee was painless with normal ROM. This girl was able to play tennis and had no difficulties cycling

MPFL reconstruction was performed in all patients, most commonly using gracilis graft (Table 1). The chondral or osteochondral fragment was either fixed (*n* = 7) (Figs. 2B and 3C–D) or removed (*n* = 4). In addition to the removal of the loose fragment, microfractures were also performed at the fracture base in two patients. In seven patients (Fig. 2B) with an osteochondral fracture, biodegradable pins were used in the fixation of the fragment. For one patient, arthroplasty included a periosteal plate containing chondral cells from the tibia, as the osteochondral fracture fragment had been in the joint for 6 years and had degenerated.

At the 6-month postoperative control examination, the knee was painless and the range of motion comparable to that of the contralateral knee in eight patients. Three patients reported pain, grumbling, snapping, and a feeling of locking in the operated knee. One patient suffered from pain on the lateral side of the patella for several months, but the pain gradually disappeared. One patient was unable to engage in sports at 6 months postoperatively owing to pain and snapping of the operated knee. MRI showed the graft to have generated hypertrophy that restricted the knee from moving normally. Soft tissue cleaning and graft resection were performed, and the knee was symptomless 6 months after reoperation.

In one patient with cartilage pin fixation, MRI at 6-month follow-up showed that a large part of the fixed cartilage tissue had disappeared/deteriorated. In another patient, the fixed osteochondral fragment had parted from the lateral facet of the patella. The loose fragment was reattached in open reduction with two pins, and plication of the medial retinaculum was performed.

At the end of follow-up, 9 of the 11 patients had Kujala scores ranging from 90 to 100. The knee performed well in most patients (Table 3). Prior to surgery, the Tegner score was 5.0 (2.7) and 5.3 (1.6) points after surgery, and KOOS-QLS was 4.1 (4.2) points at follow-up measurement. One patient had two recurrent patellar dislocations due to graft failure after MPFL reconstruction and was reoperated during the follow-up period of 2 years. One patient with a positive J-sign underwent distalization of the tibial tubercle 8 months after the fixation of the fracture combined with MPFL reconstruction.

**Table 3:** Mechanism of injury and treatment results at follow-up

Patient number	Mechanism of injury	Treatment of subchondral laminar or osteochondral fracture	Length of follow-up (years)	Recurrent dislocations	Reoperations	Kujala score		Tegner score	KOOS HRQoL (0–16)
							Before patellar dislocation	After follow-up	
1	Knee twisting	Removal	3.1	0	0	100	3	7	0
2	Knee extension	Fixation	4.5	0	0	100	1	4	1
3	Raising from kneeling	Removal	4.2	0	0	90	6	6	3
4	Straight impact with bend knee	Fixation	6.0	2	1	98	7	6	3
5	Knee twisting while jumping	Fixation	4.0	0	1	66	7	4	9
6	Running	Fixation	5.1	0	0	98	3	2	5
7	Knee twisting while jumping	Fixation	4.4	0	0	94	7	6	4
8	Knee twisting	Fixation	4.2	0	0	98	4	4	4
9	Walking	Removal	3.5	0	0	96	7	6	2
10	Knee twisting	Fixation	4.8	0	0	47	1	7	14
11	Raising the stairs	Removal	8.0	0	0	91	9	7	0
Mean (SD)			4.7 (1.3)	–	–	89 (17)	5.0 (2.7)	5.4 (1.6)	4.1 (4.2)

*KOOS* Knee and Osteoarthritis Outcome Score, *HRQoL* health-related quality of life, *SD* standard deviation

## Discussion

During the follow-up of the present series of patients with patellar dislocation, one of the 11 patients experienced recurrent patellar dislocation after fixation of chondral or osteochondral fracture and MPFL reconstruction. Moreover, one patient underwent a reoperation due to a failed graft, and another patient underwent subsequent distalization of the tibial tubercle to stabilize the patella in the trochlear groove. Nine patients had excellent scores in the outcome measures.

Nonoperative treatment is the first-line treatment for most patients with patellar dislocation [23]. However, recurrence of patellar dislocation or severe symptoms of the injured knee after primary dislocation, such as pain, locking, and movement limitation, are indications for operative treatment and MPFL reconstruction [24, 25]. Rotational profile, bony malformations [26], hip–knee–ankle angle, J-sign, and anamnesis of recurrence of patellar dislocation should be considered in the planning of treatment in elective patients. Gracilis graft is always the first choice for MPFL reconstruction. Other options, such as semitendinosus, can be used if the gracilis has already been grafted or in rare cases when the grafting of the gracilis graft fails. In patients with open growth plates, abductor magnus tenodesis is used.

Pronounced femoral valgus or torsion can be corrected with a femoral derotational osteotomy [5]. In practice, the authors do not perform lateral release except in permanent patellar dislocation situations and in cases of trochleoplasty when the lengthening of lateral capsular complex is conducted.

Over the last two decades, only small [5, 27, 28] case series and case reports (sample sizes ranging from 1 to 9) have been published on the treatment of osteochondral fractures caused by traumatic patellar dislocation. It has been approximated that 95% of patellar dislocation patients have articular cartilage injuries of the patellofemoral joint and/or patella [29]. Osteochondral fractures have been found in 39% of patellar dislocation patients of a growing age [30]. Intra-articular fractures caused by primary traumatic patellar dislocation may require operative treatment [2]. In the USA, approximately half of all patients with patellar dislocation requiring operative treatment are aged between 10 and 19 years [31]. Cartilage repair operations constitute approximately 31% of all surgical operations associated with patellar dislocation [32]. Lee et al. [5] performed microfractures at the fracture site with every adolescent patient who underwent removal of an osteochondral fracture fragment [5]. In their series, the patient-reported International Knee Documentation Committee mean outcome scores for fixation of OCF were 66 (SD, 18) and for nonfixation 76 (SD, 11.7) points. The KOOS subscale scores for quality of life were higher for the nonfixation group compared with the fixation group. In the present study, microfractures were also performed for two patients who had fragments removed. It has been suggested that attempts should be made to fix osteochondral fragments of 1 cm^2^ or larger in their original location [31]. However, the fragments in the present study were larger than 1 cm^2^ in 9 of the 11 patients. In the authors’ clinical experience, an intra-articular cartilage fragment floating in the synovial fluid can swell abundantly, while the fracture site cartilage can also potentially swell. This swelling might be due to the relatively long interval between the trauma and the repair. Thus, the fragment must be reduced to fit into the defective knee cartilage. After the fragment has been suitably reduced, fixation can be conducted with biodegradable pins, rods, or sutures [3]. If the fragment is large enough, however, fixation can be performed with biodegradable or regular screws or by combinations of these [32, 33]. In the present series of intra-articular bone or cartilage procedures, no removal of OCFs was identified. If the fragment is fragmented from the non-weight-bearing surface of the knee, it can be removed through arthroscopy or with open surgery.

Complications in the surgical treatment of OCF combined with MPFL reconstruction are rare, and patients most likely have painless, insignificant crepitation of the knee. In most cases, pain is rarely present. However, failure of the tendon graft attachment or fracture of the patella after MPFL reconstruction is possible. Fixed patellar OCF can be detached and often degenerate over time. According to the authors’ findings, the short-term outcomes of operative treatment of a subchondral laminar or osteochondral fracture with combined MPFL reconstruction were rated as good using the Kujala, the Tegner, and the KOOS HRQoL instruments. Graft failure in one patient during follow-up required re-reconstruction of the MPFL. Furthermore, the scores of two patients indicated poor outcomes. For the patients with the poorest outcomes, the authors have planned derotation osteotomy, trochleoplasty, and MPFL re-reconstruction, as the graft will be lost during the operation. Further operations require a precise assessment of alignment, the anatomical measurement of the knee, and the overall assessment of the patient’s current ability to function.

To summarize, the results of the present study indicate that favorable outcomes can be achieved in patients who undergo chondral or osteochondral fragment removal and MPFL reconstruction after primary or recurrent patellar dislocation. The limitations of the present study are the small sample size and the retrospective study design, which may lead to selection bias. As this study relied on a descriptive convenience sample and was retrospective in nature, all patients were included. Thus, it was not feasible to conduct power analyses in this study. Furthermore, no statistical tests were performed that would have needed estimation of power. Future research should therefore include a comprehensively planned randomized multicenter study on the treatment of osteochondral fractures. The authors’ experience and the results from this study can be used to provide better treatment for patients who suffer from primary or recurrent patellar dislocation with OCFs.

In conclusion, the removal or fixation of fracture fragments combined with MPFL reconstruction is a feasible option in the treatment of symptomatic osteochondral or subchondral fragments in traumatic patellar dislocation. The short-term outcomes are encouraging.

## Data Availability

The data are available from the authors on reasonable request.

## References

[1] Uimonen MM, Repo JP, Huttunen TT, Nurmi H, Mattila VM, Paloneva J (2021). Surgery for patellar dislocation has evolved towards anatomical reconstructions with assessment and treatment of anatomical risk factors. Knee Surg Sports Traumatol Arthrosc.

[2] Duthon VB (2015). Acute traumatic patellar dislocation. Orthop Traumatol Surg Res.

[3] Stefancin JJ, Parker RD (2007). First-time traumatic patellar dislocation: a systematic review. Clin Orthop Relat Res.

[4] Uimonen M, Ponkilainen V, Paloneva J, Mattila VM, Nurmi H, Repo JP (2021). Characteristics of osteochondral fractures caused by patellar dislocation. Orthop J Sports Med.

[5] Lee BJ, Christino MA, Daniels AH (2013). Adolescent patellar osteochondral fracture following patellar dislocation. Knee Surg Sports Traumatol Arthrosc.

[6] Guettler JH, Demetropoulos CK, Yang KH (2004). Osteochondral defects in the human knee: influence of defect size on cartilage rim stress and load redistribution to surrounding cartilage. Am J Sports Med.

[7] Uimonen M, Ponkilainen V, Hirvinen S, Mattila VM, Kask G, Nurmi H, Paloneva J, Repo JP (2021). The risk of osteochondral fracture after patellar dislocation is related to patellofemoral anatomy. Knee Surg Sports Traumatol Arthrosc.

[8] Spahn G, Kirschbaum S (2005). Operative treatment of deep chondral defects of the patella: results after abrasive arthroplasty and periosteal arthroplasty. Knee Surg Sports Traumatol Arthrosc.

[9] Balcarek P, Ammon J, Frosch S (2010). Magnetic resonance imaging characteristics of the medial patellofemoral ligament lesion in acute lateral patellar dislocations considering trochlear dysplasia, patella alta, and tibial tuberosity–trochlear groove distance. Arthroscopy.

[10] Dejour H, Walch G, Nove-Josserand L (1994). Factors of patellar instability: an anatomic radiographic study. Knee Surg Sports Traumatol Arthrosc.

[11] Insall J, Salvati E (1971). Patella position in the normal knee joint. Radiology.

[12] Caton J, Deschamps G, Chambat P (1982). Patella infera. Apropos of 128 cases. Rev Chir Orthop Reparatrice Appar Mot.

[13] Barnett AJ, Prentice M, Mandalia V (2009). Patellar height measurement in trochlear dysplasia. Knee Surg Sports Traumatol Arthrosc.

[14] Kadakia NR, Ilahi OA (2003). Interobserver variability of the Insall–Salvati ratio. Orthopedics.

[15] Biedert RM, Albrecht S (2006). The patellotrochlear index: a new index for assessing patellar height. Knee Surg Sports Traumatol Arthrosc.

[16] Pandit S, Frampton C, Stoddart J (2011). Magnetic resonance imaging assessment of tibial tuberosity–trochlear groove distance: normal values for males and females. Int Orthop.

[17] Dickens AJ, Morrell NT, Doering A (2014). Tibial tubercle–trochlear groove distance: defining normal in a pediatric population. J Bone Surg Am Ed.

[18] Seitlinger G, Scheurecker G, Högler R (2012). Tibial tubercle–posterior cruciate ligament distance: a new measurement to define the position of the tibial tubercle in patients with patellar dislocation. Am J Sports Med.

[19] Daynes J, Hinckel BB, Farr J (2016). Tibial tuberosity—posterior cruciate ligament distance. J Knee Surg.

[20] Kujala UM, Jaakkola LH, Koskinen SK (1993). Scoring of patellofemoral disorders. Arthroscopy.

[21] Tegner Y, Lysholm J (1985). Rating systems in the evaluation of knee ligament injuries. Clin Orthop Relat Res.

[22] Multanen J, Honkanen M, Häkkinen A (2018). Construct validity and reliability of the Finnish version of the Knee Injury and Osteoarthritis Outcome Score. BMC Musculoskelet Disord.

[23] Sillanpaa PJ, Maenpaa HM (2012). First-time patellar dislocation: surgery or conservative treatment?. Sports Med Arthrosc Rev.

[24] Askenberger M, Bengtsson Moström E, Ekström W (2018). Operative repair of medial patellofemoral ligament injury versus knee brace in children with an acute first-time traumatic patellar dislocation: a randomized controlled trial. Am J Sports Med.

[25] Longo UG, Berton A, Salvatore G (2016). Medial patellofemoral ligament reconstruction combined with bony procedures for patellar instability: current indications, outcomes, and complications. Arthroscopy.

[26] Dejour D, Le Coultre B (2018). Osteotomies in patello-femoral instabilities. Sports Med Arthrosc Rev.

[27] Nakamura N, Horibe S, Iwahashi T (2004). Healing of a chondral fragment of the knee in an adolescent after internal fixation. A case report. J Bone Surg Am Ed..

[28] Mostrom EB, Mikkelsen C, Weidenhielm L (2014). Long-term follow-up of nonoperatively and operatively treated acute primary patellar dislocation in skeletally immature patients. Sci World J.

[29] Nomura E, Inoue M, Kurimura M (2003). Chondral and osteochondral injuries associated with acute patellar dislocation. Arthroscopy.

[30] Nietosvaara Y, Aalto K, Kallio PE (1994). Acute patellar dislocation in children: incidence and associated osteochondral fractures. J Pediatr Orthop.

[31] Arshi A, Cohen JR, Wang JC (2016). Operative management of patellar instability in the United States: an evaluation of national practice patterns, surgical trends, and complications. Am J Sports Med.

[32] Walsh SJ, Boyle MJ, Morganti V (2008). Large osteochondral fractures of the lateral femoral condyle in the adolescent: outcome of bioabsorbable pin fixation. J Bone Surg Am Ed.

[33] Koëter S, van Loon C, van Susante J (2006). Lateral femoral condyle osteochondral fracture caused by a patella luxation: advantages and disadvantages of PLA fixation. Eur J Orthop Surg Traumatol.

